# Supplementary figures and images for: Opto-RhoGEFs, an optimized optogenetic toolbox to reversibly control Rho GTPase activity on a global to subcellular scale, enabling precise control over vascular endothelial barrier strength
Source: eLife. 2023 Jul 14;12:RP84364. doi: 10.7554/eLife.84364 (PMC10393062; doi:10.7554/eLife.84364)

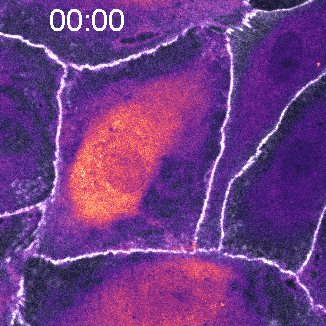

Supplement: Supplementary file 1 [file elife-84364-animation1.gif]

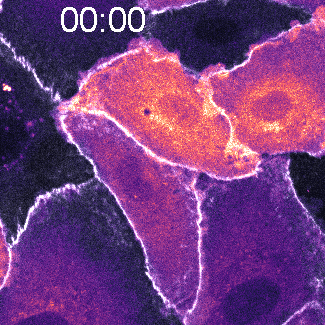

Supplement: Supplementary file 2 [file elife-84364-animation2.gif]

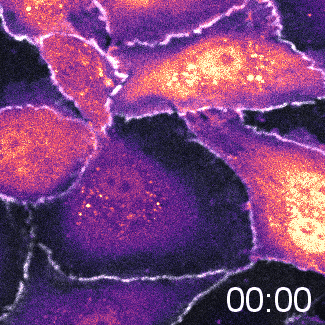

Supplement: Supplementary file 3 [file elife-84364-animation3.gif]

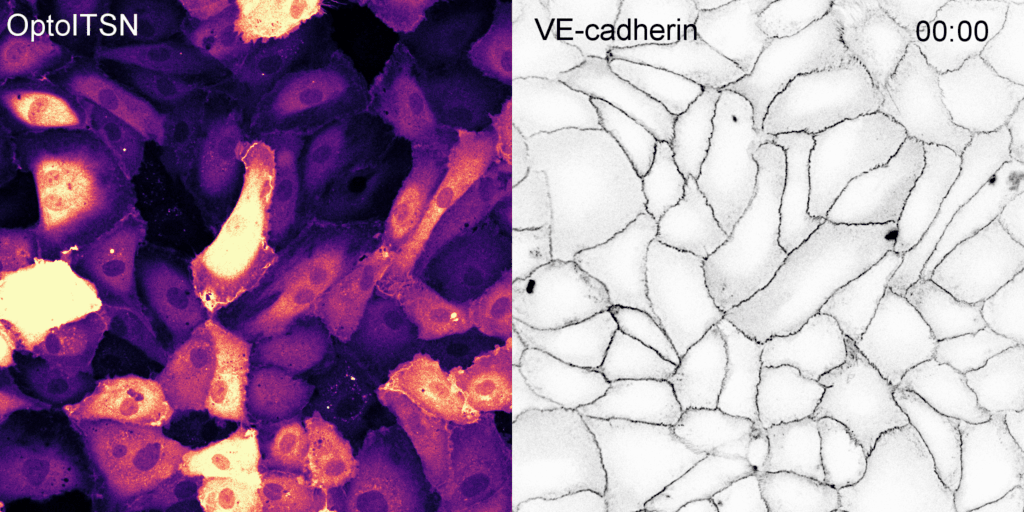

Supplement: Supplementary file 5 [file elife-84364-animation5.gif]

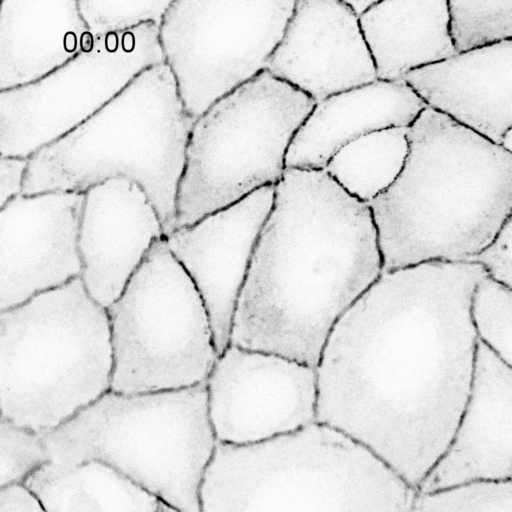

Supplement: Supplementary file 7 [file elife-84364-animation7.gif]

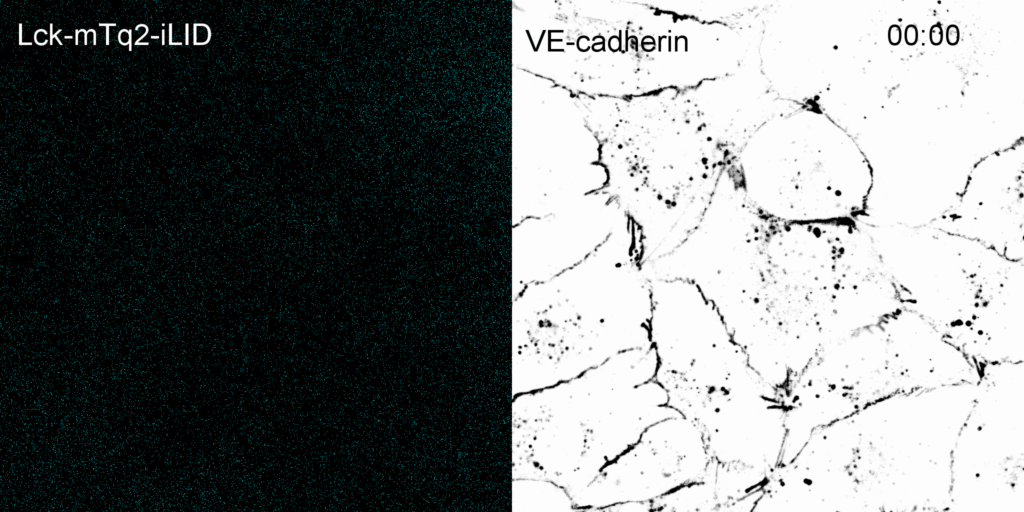

Supplement: Supplementary file 8 [file elife-84364-animation8.gif]

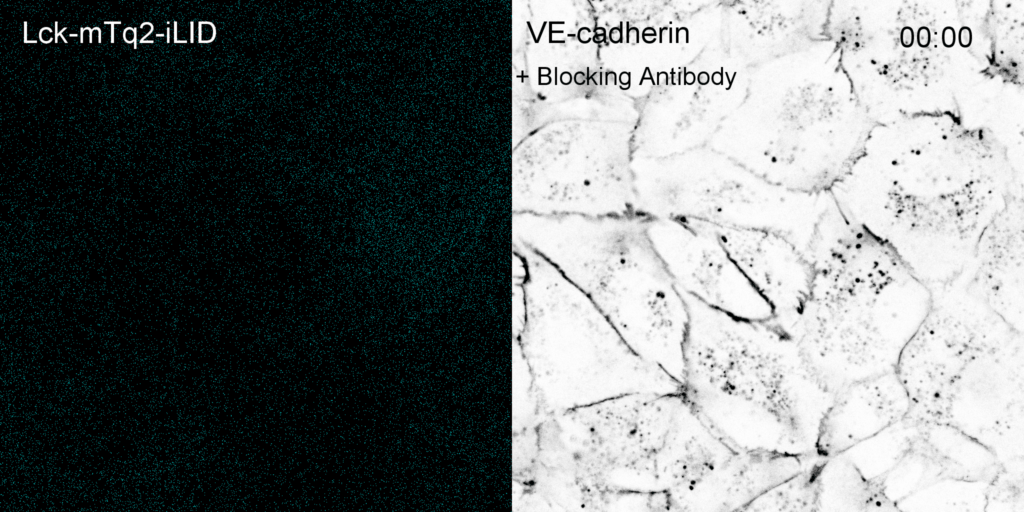

Supplement: Supplementary file 9 [file elife-84364-animation9.gif]

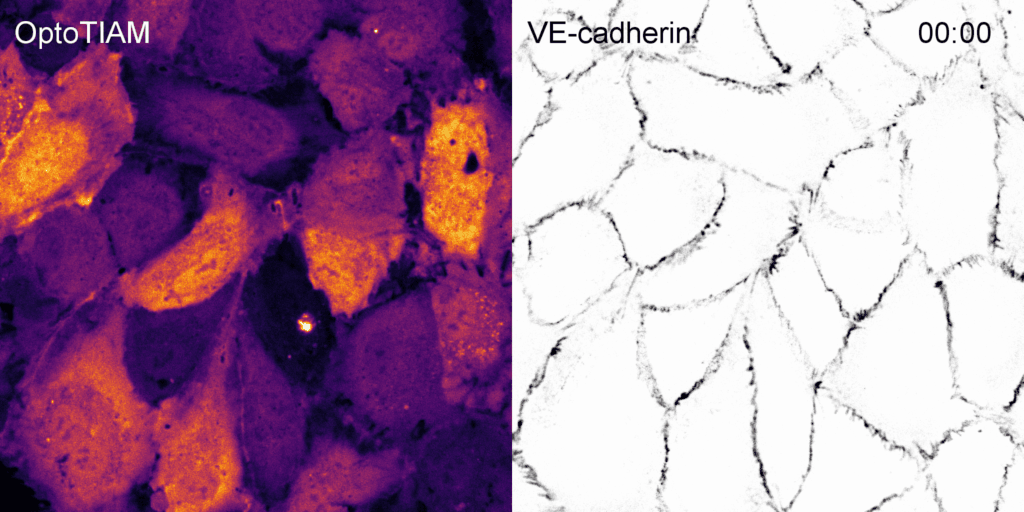

Supplement: Supplementary file 10 [file elife-84364-animation10.gif]

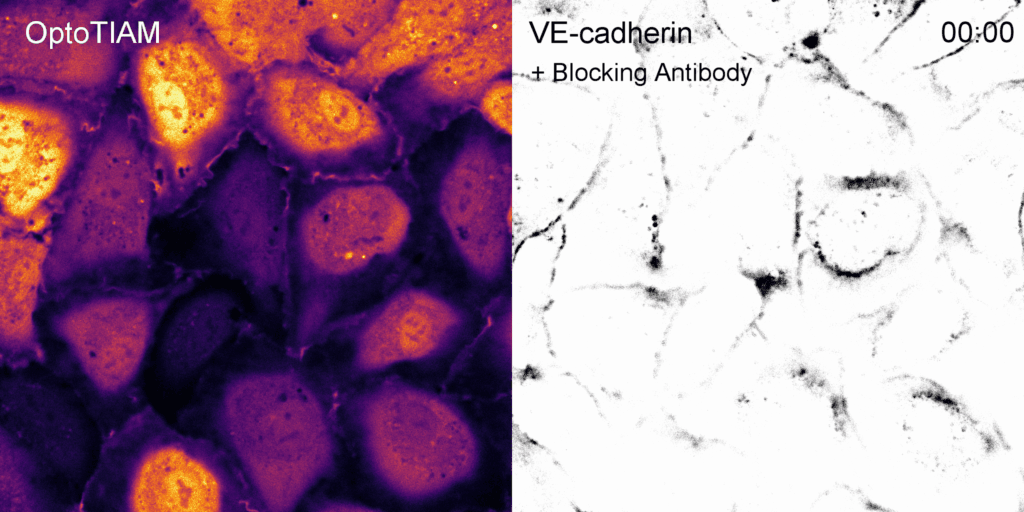

Supplement: Supplementary file 11 [file elife-84364-animation11.gif]

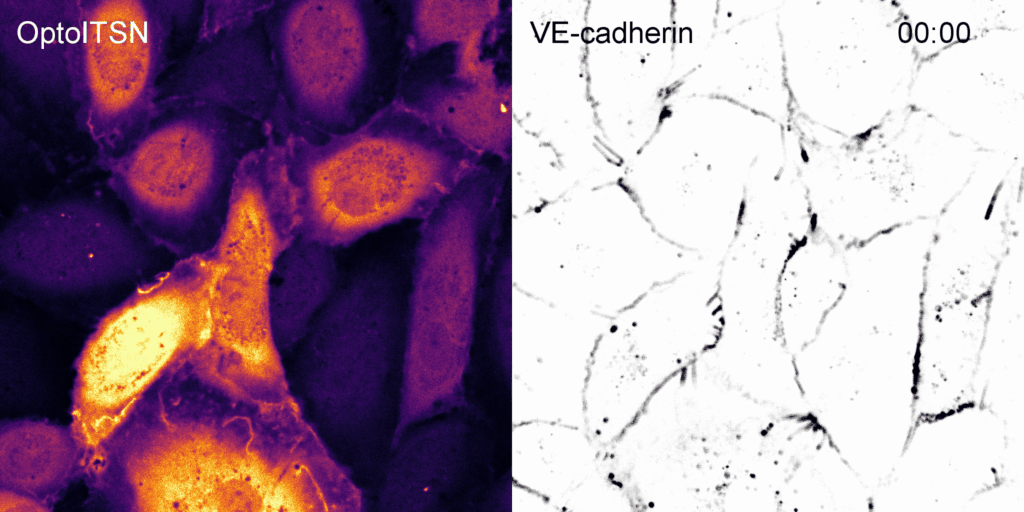

Supplement: Supplementary file 12 [file elife-84364-animation12.gif]

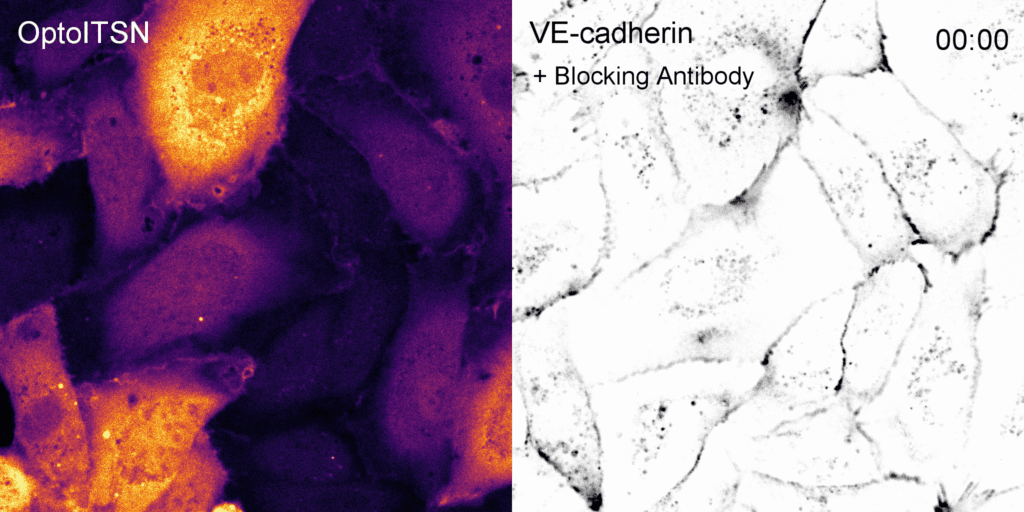

Supplement: Supplementary file 13 [file elife-84364-animation13.gif]

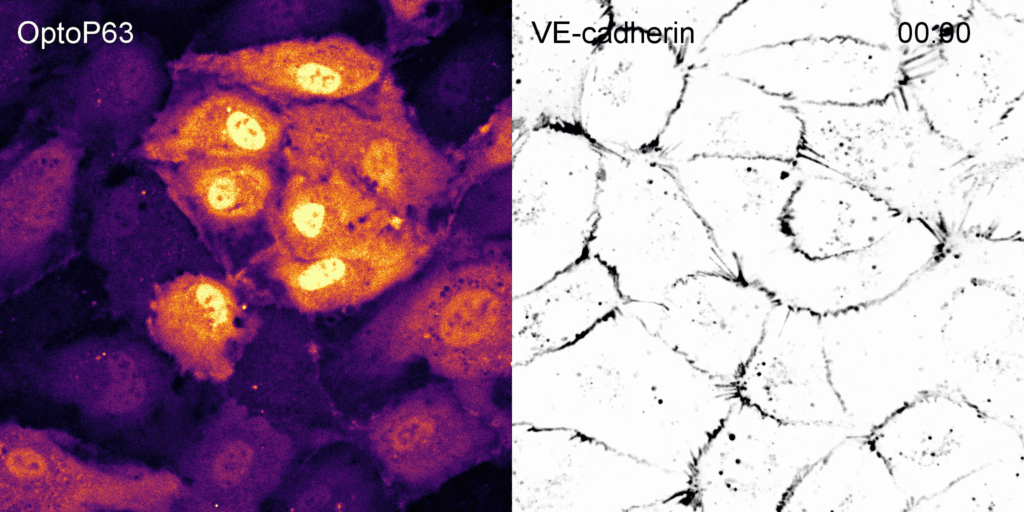

Supplement: Supplementary file 14 [file elife-84364-animation14.gif]

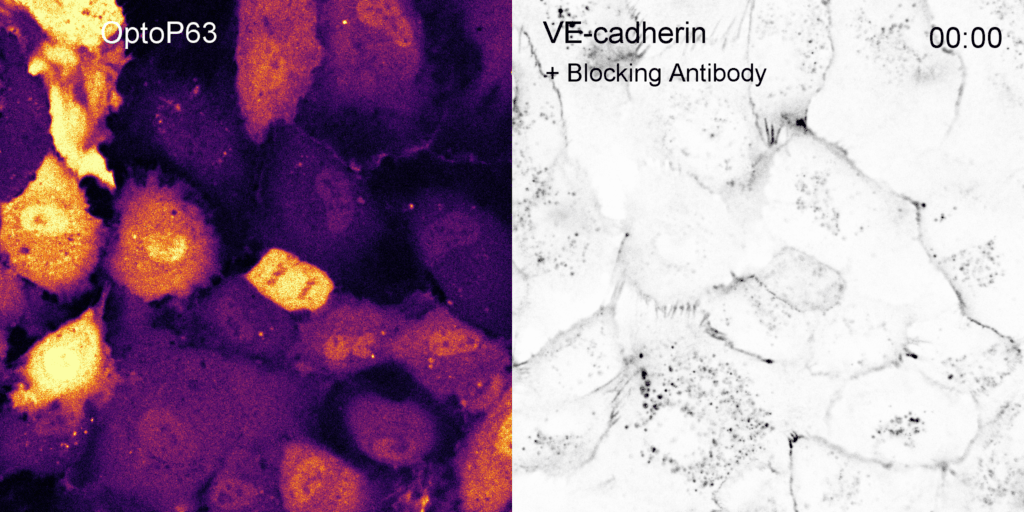

Supplement: Supplementary file 15 [file elife-84364-animation15.gif]

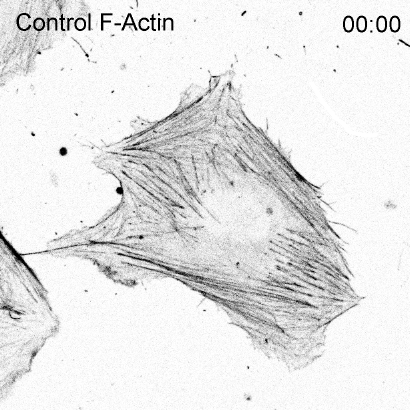

Supplement: Supplementary file 16 [file elife-84364-animation16.gif]

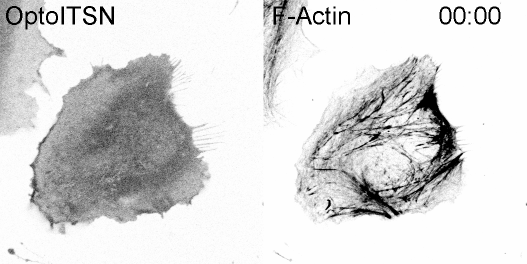

Supplement: Supplementary file 17 [file elife-84364-animation17.gif]

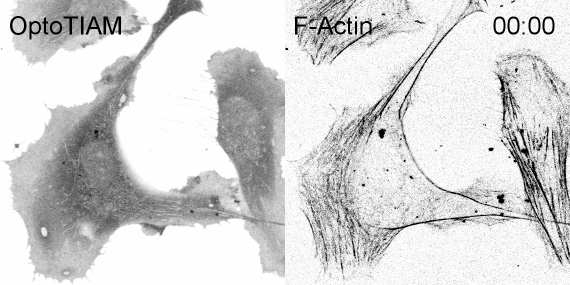

Supplement: Supplementary file 18 [file elife-84364-animation18.gif]

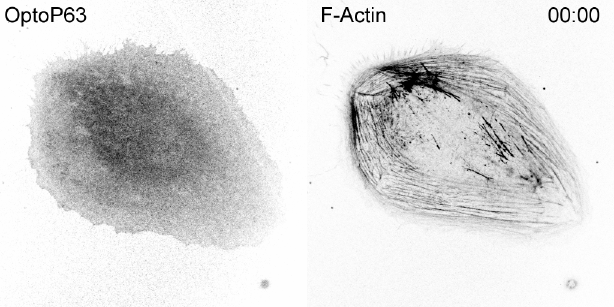

Supplement: Supplementary file 19 [file elife-84364-animation19.gif]

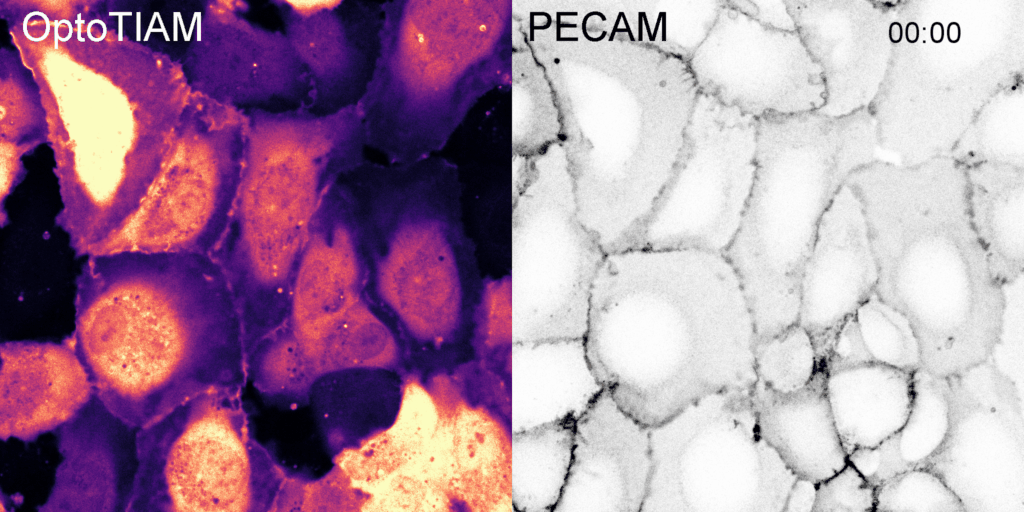

Supplement: Supplementary file 20 [file elife-84364-animation20.gif]

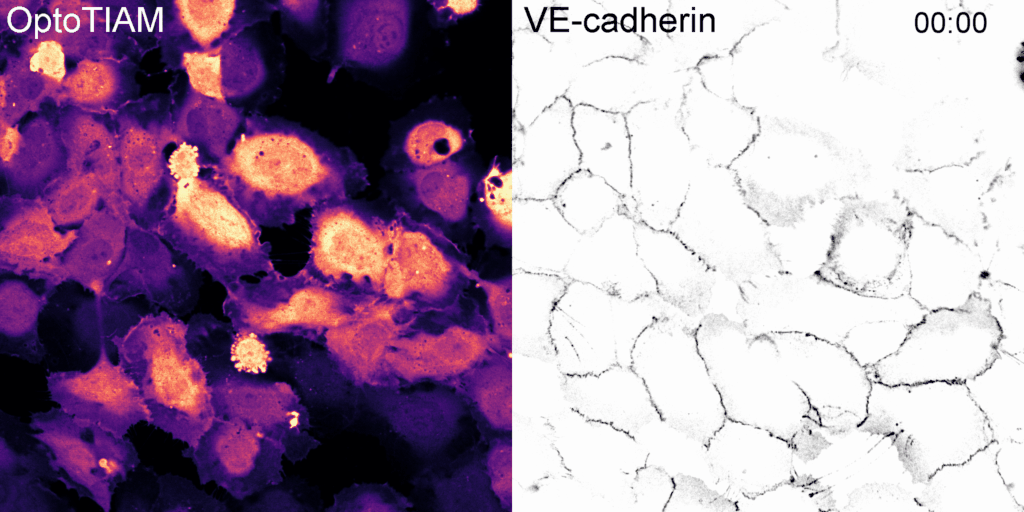

Supplement: Supplementary file 21 [file elife-84364-animation21.gif]

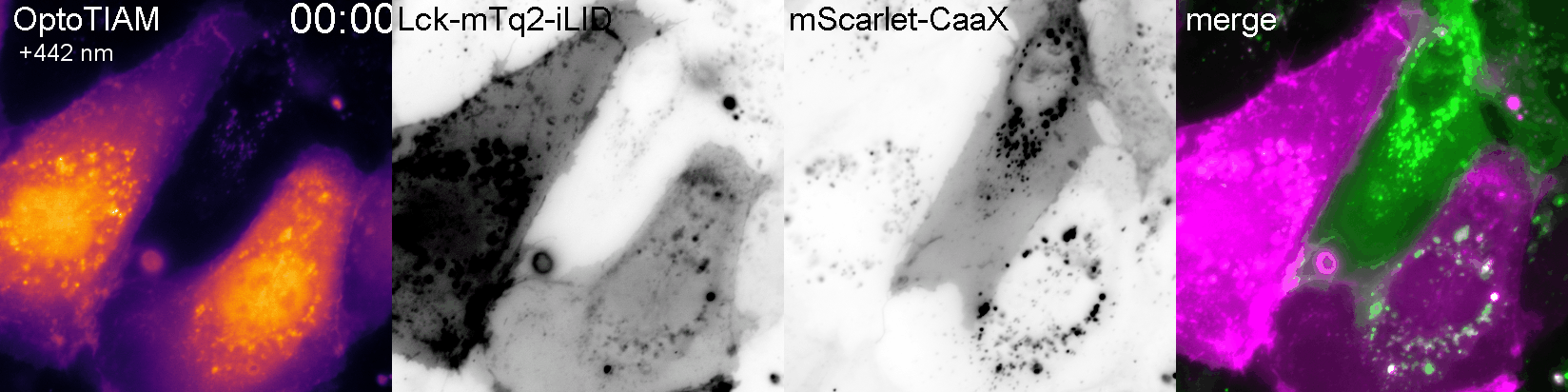

Supplement: Supplementary file 22 [file elife-84364-animation22.gif]

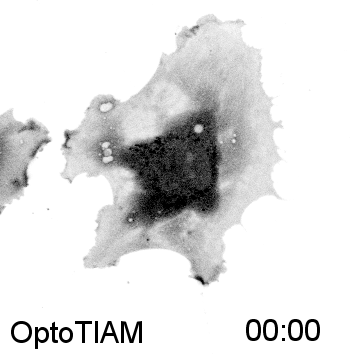

Supplement: Supplementary file 23 [file elife-84364-animation23.gif]

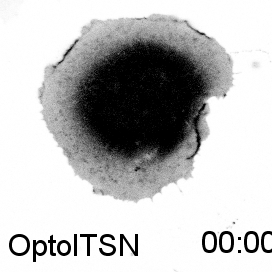

Supplement: Supplementary file 24 [file elife-84364-animation24.gif]

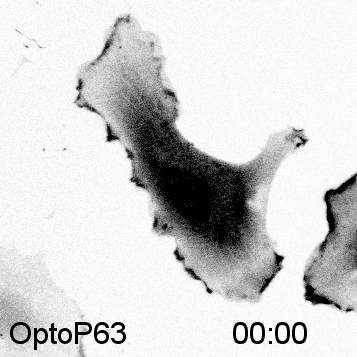

Supplement: Supplementary file 25 [file elife-84364-animation25.gif]

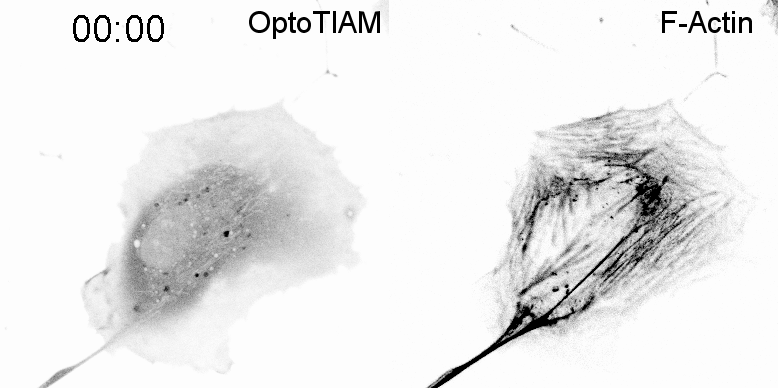

Supplement: Supplementary file 26 [file elife-84364-animation26.gif]

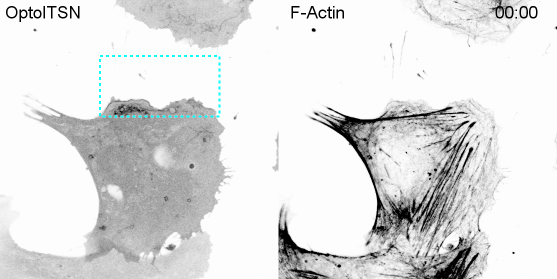

Supplement: Supplementary file 27 [file elife-84364-animation27.gif]

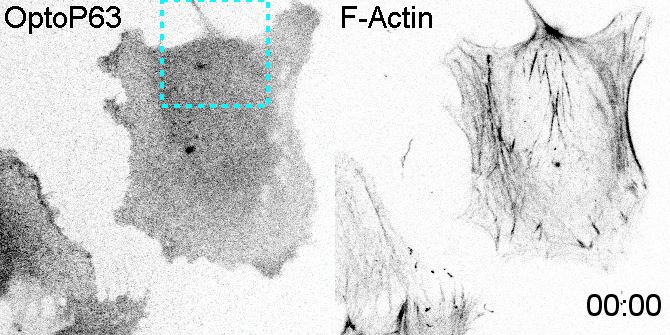

Supplement: Supplementary file 28 [file elife-84364-animation28.gif]

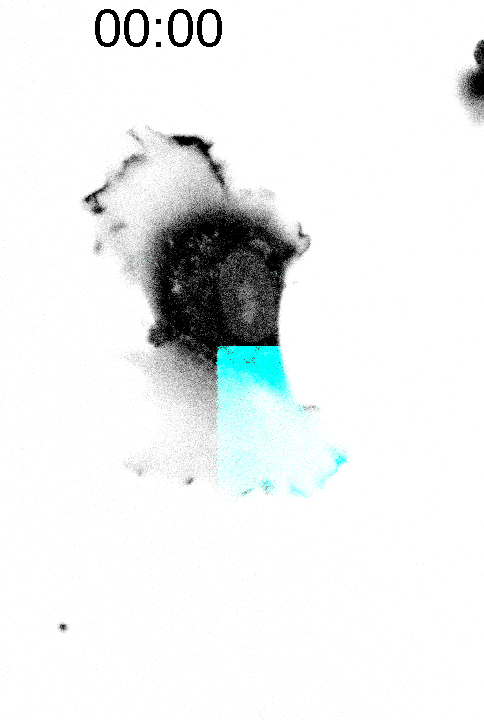

Supplement: Supplementary file 29 [file elife-84364-animation29.gif]

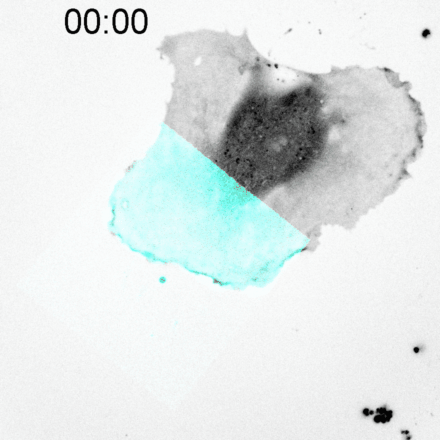

Supplement: Supplementary file 30 [file elife-84364-animation30.gif]
